# Use of Autochthonous Lactobacilli to Increase the Safety of Zgougou

**DOI:** 10.3390/microorganisms8010029

**Published:** 2019-12-22

**Authors:** Fabio Minervini, Jihen Missaoui, Giuseppe Celano, Maria Calasso, Lotfi Achour, Dalila Saidane, Marco Gobbetti, Maria De Angelis

**Affiliations:** 1 Department of Soil, Plant and Food Sciences, University of Bari Aldo Moro, 70126 Bari, Italy; 2 Laboratory of Analysis, Treatment and Valuation of Environmental Pollutants and Products, Faculty of Pharmacy, University of Monastir, 5000 Monastir, Tunisia; 3 Bio-resources: Integrative Biology & Valorization, Higher Institute of Biotechnology of Monastir, University of Monastir, 5000 Monastir, Tunisia; 4 Faculty of Science and Technology, Free University of Bozen, 39100 Bolzano, Italy

**Keywords:** Aleppo pine, seeds, juice, autochthonous lactobacilli, fermentation, antibacterial activity, antioxidant activity

## Abstract

Seeds of *Pinus halepensis* are used for preparing zgougou, a spontaneously fermented matrix giving juice and seeds debris, consumed in many Arabian countries, including Tunisia. In the same way as all the food processes based on spontaneous fermentation, zgougou hides health risks due to eventual pathogenic microorganisms and derived toxins. This study aimed at investigating the effect of the use of autochthonous *Lactobacillus paraplantarum* A1 and *Lactobacillus plantarum* A2, as fermentation starters, on the microbiological characteristics, profiles of volatile organic compounds (VOC), antibacterial and antioxidant activities of juice and seeds debris from zgougou. The starter lactobacilli inhibited undesired bacteria (e.g., *Enterobacter* and *Aeromonas*) and coccus-shaped lactic acid bacteria, as shown by culture-dependent and-independent methods. The inhibitory effect was more evident in juice than in seeds debris. Some VOC (ethanol, acetoin, phenol,2-methoxy and caryophyllene) were present at higher concentrations in juice and seeds obtained upon spontaneous fermentation, compared to the samples deriving from fermentation with lactobacilli. The latter samples were characterized by higher concentrations of acetic acid, decane, 1-nonanol, bornyl acetate and bornyl formate. In addition, they showed a wider spectrum of antibacterial activity than spontaneously fermented juice and seeds. The use of autochthonous lactobacilli did not relevantly affect the antioxidant activity of zgougou. When juice from lactobacilli-driven fermentation was used to prepare a traditional Tunisian pudding (“Assidat-Zgougou”), it improved color and odor with respect to the pudding containing juice from spontaneous fermentation. This study showed that the use, at laboratory scale, of autochthonous lactobacilli is a feasible biotechnological tool to outgrow undesired bacteria, thus improving the safety of zgougou juice. Future studies should be undertaken to confirm the observed benefits at industrial scale.

## 1. Introduction

*Pinus* (common name: pine) is the largest genus of conifers growing especially in the north of Mediterranean area. This pioneer and expansionist conifer is widely used in traditional therapeutic practice in the world and has economic importance by dint of richness of its resin and essential oil in secondary metabolites (turpentine, terpenes, phenolics, etc.,) [1,2]. In particular, seeds of *Pinus halepensis* (Aleppo pine) are traditionally used throughout Tunisia and other Arabic countries for preparing a sweet cream pudding called “Assidat-Zgougou”, and as aromatic ingredients in ice-cream and candies. The juice is commonly used as ingredient, together with white flour, sugar and, in some cases, concentrated milk, for preparing the Assidat-Zgougou on the festival of Mouled (a religious feast to celebrate the birth of the Prophet MUHAMMED “sallallahu ‘alayhi wa sallam”). The dough undergoes a cooking step until becoming a concentrated black-grey cream pudding. On the contrary, the seeds debris are not cooked, but they are usually mixed with dates and dried fruits and eaten as sweet candies. Seed debris may also be used as raw matter for extraction of essential oil. Tunisia is the only country where the tree’s black-grey seeds are specifically harvested for human consumption. People consume food items based on Aleppo pine’s seeds because they appreciate aroma and high nutritional value [3]. For instance, 100 g of Assidat-Zgougou provide nearly 275 calories [4]. In 2012, the yearly consumption of seeds was in the order of 300–320 tons, just referring to Tunisia, and is constantly increasing across years [5].

The first step of Assidat-Zgougou production is to obtain a watery mixture of ground Aleppo pine seeds, which is then subjected to a 24 h long resting step, at room temperature. During this step a fermentation occurs, which for most of cases is carried out without any addition of microbial starters. Lactic acid bacteria (LAB), one of the autochthonous components of microbiota of seeds, could play a key-role for this spontaneous fermentation. The fermented watery mixture, called “zgougou”, is subjected to coarse filtration, to separate juice from seeds debris [6,7]. Lactic acid fermentation is one of the oldest known methods for food production and/or preservation, allowing to obtain a more assorted and attractive array of food products and, at the same time, to counteract undesired microorganisms. In addition, fermentation by LAB may provide food with health-promoting properties [8,9]. For instance, antioxidant activity, linked to many chronic diseases (cancer) [9,10,11], and aging is usually enhanced by lactic acid fermentation [12,13]. However, food production relying on spontaneous fermentation represents a safety issue for consumers and is subjected to failure. Indeed, seeds and other organs from vegetables may be greatly contaminated by spore-forming pathogenic bacteria, yeasts and molds. If these undesired microorganisms prevail over LAB, the safety and sensory quality of the resulting food will be insufficient [14,15,16,17]. The use of autochthonous microorganisms could be a tool to solve the issues related to spontaneous fermentation.

Therefore, this study aimed to investigate the effect of the use of lactobacilli isolated from Aleppo pine’s seeds [18] on the microbial community, antimicrobial and antioxidant activity of fermented zgougou. In addition, the eventual effect exerted by autochthonous lactobacilli on the antioxidant activity and sensory properties of Assidat-Zgougou was also investigated.

## 2. Results

### 2.1. Selected Autochthonous Lactic Acid Bacteria for Fermentation of Aleppo Pine’s Seeds

The initial pH of the mixture of Aleppo pine’s seeds and water was ca. 5.85. After 24 h of fermentation, all the autochthonous lactic acid bacteria (LAB) decreased the pH below 5.00 (Table 1). *Lactobacillus paraplantarum* A1 and *Lactobacillus plantarum* A2 showed the lowest values of pH (ca. 4.65) and therefore were selected as starters for fermentation of zgougou.

### 2.2. Cultivable Microbiota of Juice and Seeds from Fermented Zgougou

Autochthonous *L. paraplantarum* A1 and *L. plantarum* A2, used in combination as starters, acidified the mixture of Aleppo pine’s seeds and water from an initial pH of ca. 5.85 to a final pH (after 24 h of fermentation) of ca. 4.38. Acidification was observed also for the spontaneously, non-inoculated fermented zgougou, although at a lesser (*p* < 0.05) extent. Indeed, the final pH reached the value of ca. 4.65.

**Figure 1:**
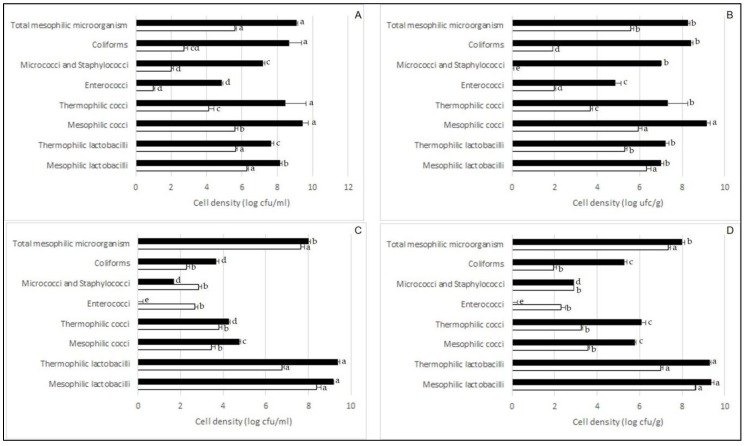
Cell densities (log CFU/mL or log CFU/g) of different bacterial groups found after 1 (white bars) and 24 (black bars) h of zgougou fermentation. (**A**) juice obtained after spontaneous fermentation (J LAB−); (**B**) seeds obtained after spontaneous fermentation (S LAB−); (**C**) juice obtained after fermentation with autochthonous lactobacilli (J LAB+); (**D**) seeds obtained after fermentation with autochthonous lactobacilli (S LAB+).

After 1 h of fermentation, presumptive coccus-shaped and rod-shaped LAB dominated the cultivable bacterial biota of non-inoculated juice (J LAB−) and seeds (S LAB−) (Figure 1, panels A and B). At the end of fermentation of the non-inoculated juice, presumptive mesophilic cocci were found at highest cell density, followed by coliforms and the other remaining groups of presumptive LAB. During fermentation, micrococci and staphylococci increased their number of ca. 5 log cycles, but remained sub-dominant compared to LAB and coliforms. High cell densities (8–9 log CFU/g) of presumptive mesophilic cocci and coliforms were found in the non-inoculated seeds after 24 h of fermentation. Other LAB groups, micrococci and staphylococci were found at sub-dominant levels. During fermentation of non-inoculated zgougou, enterococci increased their number of 3–4 log cycles, reaching a final cell density in the order of 5 log CFU/mL (juice) or g (seeds).

After 1 h of fermentation of zgougou obtained through chemical sterilization of seeds and with addition of autochthonous lactobacilli (J LAB+ and S LAB+), presumptive mesophilic lactobacilli were found at the highest cell density (8.5 log CFU/mL or g), followed by thermophilic lactobacilli (Figure 1, panels C and D). The other bacterial groups, excepted total mesophilic microorganisms, were found at values lower than 4 log CFU/mL or g. After 24 h of fermentation, lactobacilli stayed dominant, being found at numbers higher than 9 log CFU/mL or g. Presumptive coccus-shaped LAB and coliforms increased their number, especially in the seeds. On the contrary, cell density of enterococci decreased below the limit of detection after 24 h of fermentation. 

### 2.3. Culture-Independent Analysis of Microbial Community of Fermented Zgougou

The bacterial diversity of zgougou after 24 h of spontaneous or autochthonous lactobacilli-driven fermentation was described by 16S metagenetic analysis. A total of 195,453 high quality sequences of 16S rRNA gene amplicons were obtained, with an average of about 24,400 sequences per sample and an average sequence length of 537 bp. Higher values of observed species, Chao1 and Shannon indexes were found for seeds, compared to juice, regardless of the use of autochthonous lactobacilli (Table 2). The juice obtained from zgougou fermentation with autochthonous lactobacilli (J LAB+) showed lower values of all the diversity indexes than the juice obtained upon spontaneous fermentation (J LAB−). A similar trend, although with lower differences, was found also when seeds from fermentation with autochthonous lactobacilli (S LAB+) were compared to seeds from spontaneous fermentation (S LAB−).

J LAB− and S LAB− shared 12 bacterial OTUs (Table 3). *Enterobacter* sp. was one of the dominant Operational Taxonomic Units (OTUs) in both J LAB− and S LAB−. *L. plantarum* was the OTU found at the highest relative abundance in J LAB−, whereas it was subdominant in S LAB−. On the opposite, *Lactococcus raffinolactis*, subdominant in J LAB−, co-dominated bacterial community of S LAB-, along with *Enterobacter* sp. Other sub-dominant OTUs found in zgougou obtained upon spontaneous fermentation were *Aeromonas* sp. and *Acinetobacter* sp.

J LAB+ bacterial community was almost exclusively dominated by *L. plantarum*. Among the OTUs retrieved in S LAB+, *Weissella confusa*, *Pantoea* sp., and especially *Enterobacter* sp. showed the highest values of relative abundance. However, the abundance of *Enterobacter* sp. was lower (*p* < 0.05) than in S LAB−. An opposite trend was found for *Pantoea* sp. Among the sub-dominant/minor OTUs, S LAB+ harbored *L. plantarum*, and *Aeromonas* sp. and *Acinetobacter* sp. at higher and lower (*p* < 0.05) abundance, respectively, than S LAB−. Overall, in all the samples, a relative abundance ranging from ca. 13 to 37% could not be attributed to any classified bacterial taxonomic group (Table 3).

DNA extracted from fermented zgougou was also used as template for describing fungal community, using 18S rRNA high throughput sequencing analysis. However, more than 98% of detected OTUs matched with DNA sequences of vegetable origin, namely *Pinus* sp. Apart from this, ten fungal OTUs were detected, seven of which were molds (Figure S1). S LAB− was the only sample that harbored all the detected fungal OTUs.

### 2.4. VOC Profile of Zgougou 

Head Space-Solid Phase Micro Extraction-Gas Chromatography-Mass Spectrometry (HS-SPME-GC-MS) analysis was applied to characterize zgougou samples before and after 24 h of fermentation. Before fermentation, overall 57 VOCs were detected and clustered into nine different chemical classes, with one compound, namely cyclo-octone,3-butoxy, unclassified (Table S1). Most of VOCs were detected in both juice and seeds, but 12 out of 57 were found just in seeds. In detail, many aldehydes (e.g., octanal and nonanal) and all the carboxylic acids were found exclusively in seeds. Only 1-hexanol and benzyl alcohol characterized the unfermented juice, although their concentration was very low. Among the different chemical classes, terpenes (including α-pinene, β-myrcene, and d-limonene) were found at highest levels in juice and seeds before fermentation.

After fermentation, concentration of some compounds, such as decane 2,4,6 dimethyl (alkanes), α-pinene, β-myrcene, and d-limonene, decreased (Table 4). Overall, fermented juice and seeds were characterized by higher concentration of phenol,2-methoxy, compared to the samples analyzed before fermentation. In addition, during fermentation, some compounds were detected, such as ethanol, acetic acid, acetoin, and beta-pinene, which were below the detection limit in the samples before fermentation. Within juice samples, those obtained upon spontaneous fermentation of zgougou (J LAB−) showed higher concentrations of ethanol, acetoin, phenol,2-methoxy, and caryophyllene than juice obtained through fermentation with autochthonous lactobacilli (J LAB+). The latter was characterized by higher concentrations of 1-hexanol, octane,4-methyl, 2-octenal, decane, cyclopentane,1-ethenyl-3-ethyl-2-methyl, and some carboxylic (hexanoic, heptanoic, octanoic) acids. In addition, 1-nonanol, 2,4-decadienal, acetic acid, 2-octanone, 1,7,7-trimethylbicyclo [2.2.1]heptane-2,5-diol, bornyl acetate and bornyl formate were detected just in J LAB+. Within seed debris samples, those obtained upon spontaneous fermentation (S LAB−) showed higher concentration of ethanol, acetaldehyde, acetoin, phenol,2-methoxy, and caryophyllene, than seeds obtained through fermentation with autochthonous lactobacilli (S LAB+). In addition, S LAB− contained some compounds, such as 3,5-octadien-2-ol, butanal,3-methyl-, sulfurous acid, nonyl pentyl ester, phenol, (+)-alpha-gurjunene, which were not detected in S LAB+. The latter sample was characterized by higher concentration of decane, acetic acid, pentanoic acid, nonanoic acid, 2-heptanone, and bornyl acetate, compared to S LAB−. Furthermore, 11 compounds (3-butoxy-1-propanol, 1-nonanol, octane,4-methyl-, benzene acetaldehyde, dodecane,4,6-dimethyl-, butanoic acid, pentyl ester, 2-undecanone, vanillin, beta- phellandrene, alpha- farnesene, and bornyl formate) were detected just in S LAB+.

### 2.5. Correlations between Bacterial Biota and VOC

Several correlations were found, at a false discovery rate (FDR) of <0.05, between the most important bacterial OTUs and VOCs, taking into account just the VOCs found at significantly different concentrations between juice and seeds obtained upon spontaneous or autochthonous lactobacilli-driven fermentation (Table S2). In detail, *L. plantarum* was positively correlated (r ≥ 0.7) with dodecane 4,6-dimethyl and 1,7,7-trimethylbicyclo [2.2.1]heptane-2,5-Diol. *Lc. raffinolactis*, *Aeromonas* sp., and *Acinetobacter* sp. were correlated with 3,5-octadien-2-ol, acetaldehyde, butanol-3-methyl, sulfurous acid,nonyl pentyl ester, acetoin, phenol, phenol-2-methoxy, (+)-alpha-gurjunene, caryophyllene, and (−)-carvone. Positive correlations were also found between *W. confusa*, *Pantoea* sp. and 3-butoxy-1-propanol, 1-hexanol, 1-nonanol, decane, pentanoic, hexanoic, octanoic and nonanoic acids, butanoic acid pentyl ester, 2-heptanone, vanillin, beta-phellandrene, bornyl acetate, alpha-farnesene, and cyclopentane,1-ethenyl-3-ethyl-2-methyl. Finally, *Enterobacter* sp. was positively correlated with phenol-2-methoxy, caryophyllene, and (−)-carvone.

### 2.6. Antibacterial Activity of Zgougou Juice and Seeds

The juice fermented with autochthonous lactobacilli (J LAB+) showed inhibitory activity towards all the target bacteria, especially *Listeria monocytogenes, Enterococcus faecalis* and *Vibrio parahaemolyticus* (Table 5). The juice obtained upon spontaneous fermentation (J LAB−) inhibited just three target bacteria, namely *Micrococcus luteus*, *Escherichia coli*, and *Bacillus cereus*. The seeds obtained from fermentation with autochthonous lactobacilli (S LAB+) inhibited all the bacteria, except for *Aeromonas hydrophila*, whereas those from spontaneous fermentation (S LAB−) were active against *E. coli*, *L. monocytogenes*, *Ent. faecalis*, *Salmonella* Typhimurium, and *V. parahaemolyticus*.

### 2.7. Antioxidant Activity of Zgougou

**Figure 2:**
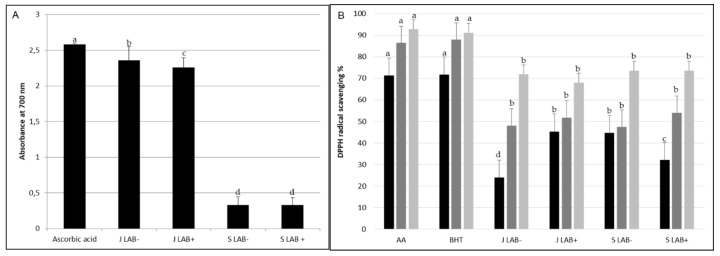
Antioxidant activity of juice (J) and seeds (S) obtained after spontaneous fermentation (LAB−) or fermentation with autochthonous lactobacilli (LAB+), as assessed through determination of ferric reducing antioxidant power (FRAP) assay after 30 min (**A**) and 2,2-DiPhenyl-1-PicrylHydrazyl (DPPH) radical scavenging activity after 0 (black bars), 20 (grey) and 120 (light grey) min (**B**). Ascorbic acid was used as positive control in both the assays. Butyl hydroxy toluene (BHT) was used as additional positive control in the DPPH assay. Within readings performed at the same incubation time, bars labelled with at least one common letter were not significantly different (*p* = 0.05).

Zgougou juice had higher ferric reducing antioxidant power (FRAP) than the seeds, regardless of the inoculation of autochthonous lactobacilli (Figure 2A). When scavenging activity against 2,2-DiPhenyl-1-PicrylHydrazyl (DPPH) was used for estimating antioxidant activity, the activity increased depending on the incubation time applied in the assay, reaching the highest value after 120 min (Figure 2B). Overall, antioxidant activity of the zgougou juice and seeds was ever lower than ascorbic acid and BHT, used as positive controls. Significant differences among the samples were found only after 20 min of incubation. In detail, juice from fermentation with lactobacilli (J LAB+) and seeds obtained with spontaneous fermentation (S LAB−) showed higher DDPH scavenging activity than the two other samples. Taken together, these results would indicate that J LAB+ had the highest antioxidant activity (FRAP and DPPH tests). 

**Figure 3:**
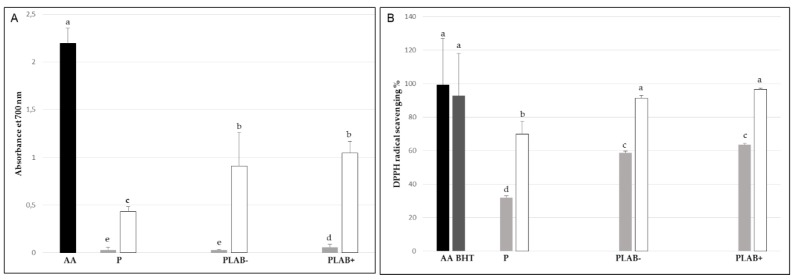
Antioxidant activity of ethanolic extracts of pudding prepared using fermented (P LAB−, spontaneously, or, P LAB+, inoculated with autochthonous lactobacilli) zgougou juice, as assessed through determination of FRAP assay after 30 min (**A**) and DPPH radical scavenging activity after 120 min (**B**). Pudding P, produced without addition of fermented zgougou juice, was used as control. Ascorbic acid was used as positive control. BHT was used as additional positive control in the DPPH assay. The extract of each pudding was tested at 100 g/L (grey bars) or 500 g/L (white). Bars labelled with at least one common letter were not significantly different (*p* = 0.05).

Antioxidant activity of puddings prepared using juice of Aleppo pine’s seeds increased depending on the concentration (100, 200, 300, 400 and 500 g/L) of ethanolic extract used. The puddings showed higher FRAP value than the control pudding, produced without addition of zgougou juice (Figure 3A). At a concentration of 500 g/L, this did not depend on the use of autochthonous lactobacilli. However, at a lower concentration (100 g/L), the pudding prepared using J LAB+ had higher (*p* < 0.05) FRAP value than the pudding with J LAB− and the control pudding. Results of DPPH radical scavenging activity confirmed those obtained using the FRAP assay, except for the concentration 100 g/L (Figure 3B). At this concentration, both puddings prepared with fermented juice had higher antioxidant activity than the control pudding. 

### 2.8. Sensory Analysis of Assidat-Zgougou Puddings 

**Figure 4:**
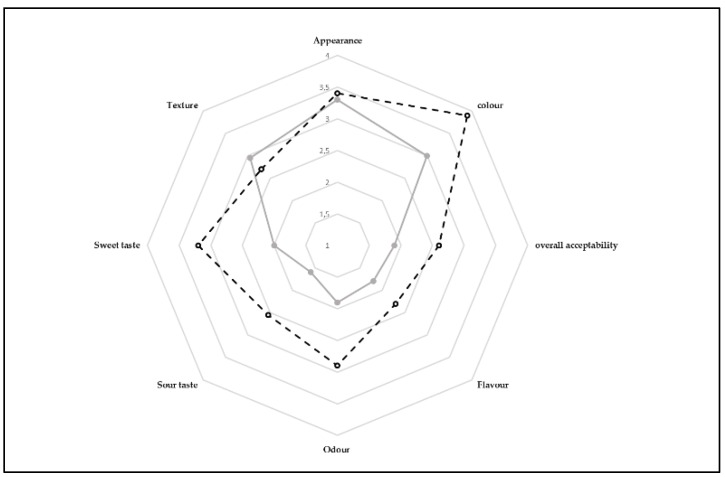
Sensory analysis of pudding prepared using juice from spontaneously fermented (P LAB−, ―•―) or fermented with autochthonous lactobacilli (P LAB+, ---◦---) zgougou.

The pudding prepared with juice from zgougou fermented with autochthonous lactobacilli (P LAB+) was rated with higher scores than the pudding prepared with juice from spontaneous fermentation (P LAB−) (Figure 4). The only exceptions were for appearance and texture, which were not significantly different between P LAB+ and P LAB−. 

## 3. Discussion

Seeds of *Pinus halepensis* are used for preparing zgougou, a spontaneously fermented matrix giving juice and seeds debris, consumed in many Arabian countries, including Tunisia [1,2,4]. Similarly, to all the food processes based on spontaneous fermentation, zgougou hides health risks due to eventual pathogenic microorganisms and derived toxins [19]. In this study, two lactobacilli, previously isolated from zgougou [18], were used as combined starters for driving fermentation, aiming especially to standardize the process and to inhibit the growth of undesired bacteria. The effect of these starters on microbiota of zgougou was evaluated by comparing the results of culture-dependent and -independent analyses, performed on juice and seeds obtained after lactobacilli-driven or spontaneous fermentation. Culture-dependent analyses after 1 h of fermentation showed that the basic bacterial biota of zgougou was mainly represented by LAB, regardless of the use of starter lactobacilli. Spontaneously fermented fruits and vegetables normally harbor LAB [20,21]. As expected, the addition of autochthonous strains belonging to the *L. plantarum* group in our zgougou samples favored mesophilic lactobacilli over the other LAB groups. During fermentation, coccus-shaped LAB and coliforms increased their number. However, the highest increases were found in spontaneously fermented zgougou samples (J LAB− and S LAB−). In addition, in the latter samples enterococci, micrococci and staphylococci increased during fermentation. On the opposite, these bacterial groups remained constant or decreased after 24 h of fermentation with autochthonous lactobacilli (J LAB+ and S LAB+), suggesting that the starter lactobacilli exerted a target-dependent inhibitory effect towards undesired bacterial groups. The results of culture-dependent analyses indicated that the decrease of pH observed in the spontaneously fermented zgougou was due to the acidifying activity of LAB, including enterococci, naturally present in the Aleppo pine’s seeds. As expected, the acidification of zgougou was higher when autochthonous lactobacilli were used as starters. In agreement with our results, an inhibitory effect of autochthonous LAB strains (belonging to *L. plantarum*, *Leuconostoc mesenteroides*, and *Pediococcus pentosaceus*) towards coliforms was found during fermentation of carrots and French beans [22].

Results from 16S metagenetic analysis of bacterial biota through Illumina MiSeq partially confirmed the results of culture-dependent analyses. In detail, *Enterobacter* sp., belonging to the group of coliforms, and *Lc. raffinolactis* were dominant in spontaneously fermented zgougou samples. On the contrary, the use of autochthonous lactobacilli, possibly in combination with chemical sterilization of raw matter (seeds), inhibited growth of *Lc*. *raffinolactis* and/or inactivated this LAB during fermentation. Similarly, to the results of this study, the use of protective lactobacilli strains (belonging to *L. plantarum* and *Lactobacillus rhamnosus*) inhibited coccus-shaped LAB (lactococci and streptococci) during cheese-making [23]. *Lc. raffinolactis* has been isolated especially from dairy environment (raw milk and ripening cheese), but rarely from vegetables [24]. The role of this LAB in food has not been elucidated so far. Inactivation/inhibition of *Enterobacter* sp. occurred in juice obtained from zgougou fermentation with autochthonous lactobacilli and, at a much lesser extent, in seeds. *Enterobacter* sp. has been reported as producer of biogenic amines [25]. In addition, it may occasionally cause food-borne infections [26]. *L*. *plantarum* dominated the bacterial biota of fermented juices, especially, as expected, when autochthonous lactobacilli were used as starters. In the seeds, it was sub-dominant, but was harbored at higher abundance in those obtained upon zgougou fermentation with lactobacilli. Among the sub-dominant/minor OTUs in the seeds, *Aeromonas* sp. and *Acinetobacter* sp. seemed to be controlled over by the use of autochthonous lactobacilli. *Aeromonas* sp. includes species causing food-borne infections [27]. *Acinetobacter* sp. includes food spoiler bacterial species [28], *Acinetobacter baumannii*, responsible for nosocomial infections [29], and other species probably responsible for food-borne diseases [30]. Unexpectedly, *Pantoea* sp. seemed to be favored, in the seeds’ debris, by the use of lactobacilli as starters for fermentation. However, we have to take into account the effect of chemical sterilization, which was carried out exclusively in the seeds destined to lactobacilli-driven fermentation. This treatment probably acted just on the surface of seeds and, therefore, did not inactivate *Pantoea* sp., which is frequently reported as endophytic bacterial genus [31]. On the contrary, other bacterial species harbored on the surface layers of seeds were inactivated by sterilization.

Fermentation of zgougou caused more complex VOC profiles of both juices and seeds, containing ca. 20 compounds, which had not been detected before fermentation. Some compounds, such as ethanol, acetic acid and acetoin, clearly derived from energy metabolism of bacteria. Besides being an end-product of fermentation of carbohydrates, a relevant amount of ethanol can participate in esterification of carboxylic acids, generating ethyl acetate (from acetic acid and ethanol), ethyl ester of hexanoic acid, and ethyl ester of octanoic acid [32]. Esters confer pleasant, sweet, fruity flavors to fermented food [32] and were found in rice fermented with *Aspergillus oryzae* or *Rhizopus oryzae* [33]. Accordingly, in the current study, some esters (e.g., ethyl acetate in juice, butanoic acid pentyl ester) were detected just after fermentation. Acetic acid, at relatively low concentrations, may act as flavor enhancer in fermented food, such as *obushera* obtained from spontaneous or starter-driven sorghum fermentation involving LAB and yeasts [34]. Acetoin, a yogurt-like odorant, was found at relatively high concentrations in rice fermented with *Lactobacillus fermentum* [33]. In the current study, zgougou fermentation differently affected concentrations of terpenes, which are intrinsic VOC for Aleppo pine’s organs (including cones, bringing seeds) and show antioxidant and antimicrobial activity [35]. Some terpenes, such as limonene, may be used as flavoring and antioxidant additives in food [36].

In the current study, ethanol, acetoin, phenol,2-methoxy and caryophyllene were present at higher concentrations in juice and seeds obtained upon spontaneous zgougou fermentation, compared to the samples deriving from fermentation with autochthonous lactobacilli. Caryophyllene decreased in fermented dry sausage with increasing doses of immobilized cells of *Lactobacillus casei* [37]. The use of autochthonous lactobacilli for zgougou fermentation caused an increase of acetic acid, decane, 1-nonanol, bornyl acetate and bornyl formate in both juices and seeds. Acetic acid may be produced upon various fermentations, including hetero-lactic fermentation. *L*. *plantarum* may operate hetero-lactic fermentation, depending on the presence of pentoses, whereas *Weissella* sp. performs hetero-lactic fermentation, regardless of the type of carbohydrates in the ecosystem [38]. 

Fermented juices and seeds from fermented zgougou showed antibacterial activity, probably resulting from a combination of compounds naturally contained in Aleppo pine’s seeds and products of LAB metabolism. Essential oil extracted from Aleppo pine’s seeds contains several antimicrobial terpenes, such as α-pinene, β-myrcene, and caryophyllene [2], detected also in the zgougou samples analysed in this study. LAB may release a wide array of antimicrobial compounds, including organic acids, diacetyl and bacteriocins [39]. In this study, both juice and seeds from zgougou fermented with autochthonous lactobacilli showed a wider spectrum of antibacterial activity than spontaneously fermented juice and seeds. This may be attributed to the capacity of *L. plantarum*, here used as starter for fermentation, to produce, besides lactic and acetic acids, 2-hydroxy-3-phenyl propionic acid (*alias* phenyllactic acid) [40], and bacteriocins named plantaricins [41]. 

Zgougou fermentation produced seeds and, especially, juices endowed with antioxidant activity, regardless of the use of autochthonous lactobacilli. Zgougou juice contained, among VOCs, α-pinene, β-myrcene, and caryophyllene, which were also detected in the essential oil of the seeds and showed antioxidant activity [42]. In addition, phenols, such as phenol-2-methoxy, found in the juices considered in this study could play an important role in antioxidant activity, because they act as effective hydrogen donors [43].

When juices were used to prepare puddings similar to Assidat-Zgougou, they showed higher antioxidant activity than a control pudding obtained without fermented juice from Aleppo pine’s seeds. Antioxidant activity of a popular custard pudding dessert was attributed to compounds derived from Maillard reaction occurring, during cooking, between psicose, a rare hexose, and free amino groups of proteins [44]. The use of autochthonous lactobacilli for obtaining the juice used as ingredient of the pudding (P LAB+) improved color and odor with respect to the pudding produced with spontaneously fermented juice. The highest score for odor, received by P LAB+, could be linked to higher alcohols, such as 1-hexanol, described as having green grass, flowery, woody, mild, sweet odor, and 1-nonanol, characterized by orchid odor. In addition, it contained bornyl formate, giving green odor. However, P LAB+ contained also higher concentrations of some carboxylic acids, giving cheesy smells, and 2-octanone, associated to soapy feeling.

## 4. Materials and Methods

### 4.1. Microorganisms, Culture Conditions and Acidification Capacity

Fourteen lactic acid bacteria (LAB) previously isolated from fermented zgougou [18] were used in this study: *L. paraplantarum* A1, *L. plantarum* A2 and A3, *Ent. faecalis* A4, A5, A6, A7, A8, A9, A10, A11, A12, A13, and A14. De Man- Rogosa-Sharpe (MRS) and M17 broth media (both from Oxoid, Basingstoke, Hampshire, UK) were routinely used for culturing (24–48 h, 37 °C) lactobacilli and enterococci, respectively. The LAB strains were screened for their acidification capacity during fermentation (24 h, 25 °C) of juice of Aleppo pine’s seeds. To this aim, bacterial cells were recovered by centrifugation (11,000× *g*, 4 °C, 20 min) from a 24-h-old liquid culture, washed with sterile saline (NaCl 9 g/L) solution and added (8 log CFU/g) in a mixture of ground seeds (100 g) and tap water (150 mL). The pH of the mixture was measured, before and after fermentation, by direct insertion of a FoodTrode (Hamilton, Bonaduz, Switzerland) electrode. The most acidifying strains were selected for the production of zgougou.

### 4.2. Protocol of Production of Zgougou 

**Figure 5:**
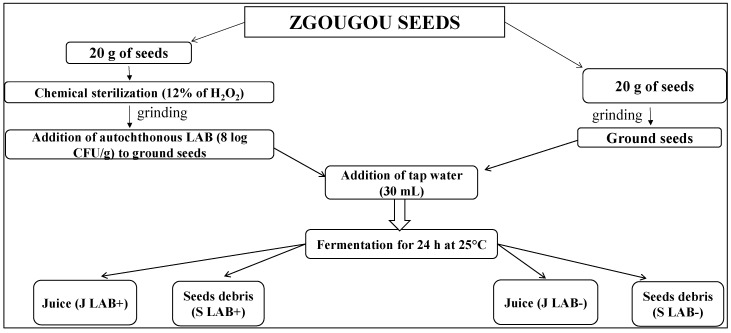
Schematic protocol applied to obtain juice (J) and seeds debris (S) from zgougou fermented with autochthonous lactobacilli (J LAB+, S LAB+) and from non-inoculated, spontaneously fermented zgougou (J LAB−, S LAB−).

Aleppo pine’s seeds (lipids, 43.3%; total carbohydrates, 25.7%; crude proteins, 22.7%; ash, 8.3% of dry matter) were harvested on December 2018 from Djabal Awladbou Ghanem, Kasserine (Foussana, Tunisia). Two batches of Aleppo pine’s seeds (20 g of each sample) were ground by coffee mill, and then 30 mL of tap water were added and mixed together (Figure 5). Before grinding, one of the two batches had been sterilized by merging seeds in a H_2_O_2_ solution (12%). Chemical sterilization was necessary because during preliminary tests the pH of the fermented matrix was ever higher than 4.5. Before incubation, the chemically sterilized seeds were inoculated with a combination of autochthonous *L. paraplantarum* A1 and *L. plantarum* A2, each at an initial cell density of ca. 8 log CFU/g, prepared as described above. The other batch was used to produce a mixture of seeds and water, which was not inoculated. Both mixtures were fermented at 25 °C for 24 h, under occasional manual stirring. After 1 h and 24 h of fermentation, aliquots of the mixtures were aseptically collected and separated by manual sifting (screening), to obtain: (i) juice (J LAB+) and seeds debris (S LAB+) from zgougou started with autochthonous lactobacilli; and (ii) juice (J LAB−) and seeds debris (S LAB−) from non-inoculated zgougou. 

### 4.3. Cultivable Microbiota

Microbiological analyses were carried out using culture media and supplements purchased from Oxoid. One milliliter of juice or a homogeneous suspension of seeds (10 g with 90 mL of saline sterile solution) were serially diluted and plated, using the following agar media and incubation conditions: Plate Count (30 °C, 24 h, for total mesophilic microorganisms), Violet Red Bile Glucose (37 °C, 24 h, for total coliforms), Baird Parker supplemented with egg yolk and tellurite (37 °C, 24–48 h, for staphylococci and micrococci), Slanetz and Bartley (37 °C, 24 h, for enterococci), M17 (30 °C or 42 °C, for mesophilic or thermophilic coccus-shaped LAB, respectively, 48 h), and MRS (30 °C or 42 °C, for mesophilic or thermophilic rod-shaped LAB, respectively, 48 h).

### 4.4. Culture-Independent Analysis of Bacterial and Fungal Microbiota

Total DNA was extracted from juice and seeds fermented (spontaneously or with the addition of autochthonous lactobacilli) for 24 h. Before extraction, 500 mg of seeds debris were homogenized with 500 μL of sterile saline solution. DNA was extracted starting from 500 mg of homogenized seeds or juice, using the FastDNA Spin Kit for Soil (MP Biomedicals, Illkrich, France), according to the manufacturer’s instructions [45]. Concentration and quality of DNA was assessed through spectrophotometric measurement, using the NanoDrop ND- 1000 (Thermo Fisher Scientific Inc, Wilmington, USA).

DNA was used as a template for high throughput sequencing analysis of bacterial and fungal diversity, using the Illumina 2 × 300 bp paired-end MiSeq platform, at Research and Testing Laboratory (RTL) Genomics (Lubbock, Texas). PCR were run according to internal protocols of RTL, using primers 28F/519R [46], targeting the V1-V3 region of 16S rRNA of Bacteria, and primers funSSUF/funSSUR [47], and targeting the ribosomal Small Subunit of Eukarya. Sequenced reads were processed as follows: (i) merged through the PEAR Illumina paired-end read merger [48]; (ii) chimeras were removed through the UCHIME software [49]; (iii) sequences were aligned using the USEARCH global alignment algorithm [50]; and (iv) OTUs were selected through the UPARSE OTU selection algorithm [51]. OTUs were identified using a NCBI database containing high quality sequences.

### 4.5. VOCs Analyses

One gram of samples, added of 10 µL of internal standard solution (2-nonane, final concentration 100 ppm), were placed into 20 mL glass vials and sealed with polytetrafluoroethylene (PTFE)-coated silicone rubber septa (20 mm diameter) (Supelco, Bellefonte, PA, USA). In order to obtain the best extraction efficiency, the HS-SPME micro-extraction procedure was performed as described in [52], with slight modifications. To keep the temperature constant during analysis, the vials were maintained on a heater plate (CTC Analytics, Zwingen, Switzerland) and the extraction was carried out by CombiPAL system injector autosampler (CTC Analytics). The sample was equilibrated at 60 °C for 20 min under gentle stirring (150 rpm). At the end of sample equilibration, a conditioned 50/30 μm DVB/CAR/PDMS fibre (Supelco, Bellefonte, PA, USA) was exposed to headspace at same temperature for 30 min to adsorb VOC. The extracted compounds were desorbed in splitless mode for 3 min at 220 °C and analysed through a Clarus 680 (PerkinElmer, Waltham, MA, USA) gas-chromatography system equipped with a capillary column Rtx-WAX column (30m × 0.25mm i.d., 0.25 μm film thickness) (Restek, Bellefonte, PA, USA). The column temperature was set initially at 35 °C for 8 min, then increased to 60 °C at 4 °C/min, to 160 °C at 6 °C/min and finally to 200 °C at 20 °C/min and held for 15 min [53]. Helium was used as the carrier gas at flow rate of 1 mL/min. The analyses lasted 50 min. The single quadrupole mass spectrometer Clarus SQ 8C (Perkien Elmer) was coupled to the gas chromatography system. The source and transfer line temperatures were kept at 250 and 230 °C, respectively. Electron ionization masses were recorded at 70 eV in the mass-to-charge ratio (m/z) interval 34–350. The GC-MS generated a chromatogram with peaks representing individual compounds. Each chromatogram was analyzed for peak identification using the National Institute of Standard and Technology (NIST) 2008 library. A peak area threshold of >1,000,000 and 90% or greater probability of match was used for VOC identification, followed, when necessary, by manual visual inspection of the fragment patterns. Compounds were quantified in terms of arbitrary area units.

### 4.6. Antibacterial Activity 

The antibacterial activity of zgougou juice and seeds was tested using the well diffusion assay [9]. Before the test, the fermented juice was centrifuged (10,000 rpm, 10 min, 4 °C) and the supernatant collected and filtered (0.22 μm). Water-soluble extract of seeds was obtained by homogenizing (3 min, Bag Mixer 400P, Interscience, Noem, France) 1 g of seeds with 4 mL of demineralized water. After centrifugation (in the same conditions as above), the supernatant was collected and filtered.

The following target species were used in the assay: *Staphylococcus aureus* ATCC 25923, *Staphylococcus epidermidis* CIP 106510, *M. luteus* NCIMB 8166, *E. coli* ATCC 35218, *L. monocytogenes* ATCC 19115, *Ent. faecalis* ATCC 29212, *Salmonella enterica* subsp. *enterica* serovar Typhimurium ATCC 1408, *Bacillus cereus* ATCC 11778, *V. parahaemolyticus* ATCC 17802, and *A. hydrophila* ATCC 7966. After culturing the bacteria in nutrient broth at 37 °C for 24 h, plates of Nutrient agar were singly inoculated, through spread technique, with 100 μL of dilutions (with NaCl 9 g/L sterile solution) of cultures, in order to get a final absorbance, at 600 nm, of 0.3 (*St. aureus*, *St. epidermidis*, *E. coli, L. monocytogenes*, *S*. Typhimurium, *B. cereus*) and 0.6 (*M. luteus, Ent. faecalis, V. parahaemolyticus,* and *A. hydrophila*) UA. The juice/seeds extract was loaded (100 μL) in wells of 5 mm diameter. After 60 min of incubation at 4 °C, plates were incubated at 37 °C. After 24–48 h, the antibacterial activity was assessed by measuring the diameter of inhibition zone (clear zone) around the well. Chloramphenicol (0.1 g/L) and sterile demineralized water were used in the assay as positive and negative controls, respectively.

### 4.7. Determination of Antioxidant Activity

Antioxidant activity was measured on juice and seeds obtained after 24 h of fermentation (spontaneous or inoculated with autochthonous lactobacilli), as well as on zgougou pudding (obtained using spontaneously or lactobacilli-driven fermented juice of Aleppo pine’s seeds) and pudding obtained without addition of fermented juice (control). For determination of antioxidant activity of seeds debris obtained after 24 h of fermentation, methanolic extract was prepared mixing 3 g of seeds and 30 mL of methanol (80%, *v/v*). After keeping this mixture under nitrogen flux for 5 min at 20 °C, the mixture was centrifuged (4032× *g*, 20 min, 4 °C) and the supernatant was subjected to analysis [54]. For determination of antioxidant activity of puddings, ethanolic extract was prepared mixing 10 g of pudding and 20 mL of absolute ethanol (99.5%, *v/v*) to form a fine smooth slurry [55]. After centrifugation (3000× *g*, 10 min, 4°C) the supernatant represented the zgougou pudding extract (concentration of 500 g/L). Dilutions of the supernatant were made at various concentrations (100, 200, 300, 400 g/L) with 75% ethanol.

Antioxidant activity was estimated in terms of FRAP and scavenging activity towards the radical DPPH. For assay of FRAP, 200 µL of sample (juice/methanolic extract of seeds/ethanolic extract of pudding) were mixed with 200 µL of 0.2 M sodium phosphate buffer (pH 6.6) and 200 µL of potassium ferricyanide (1%, *w/v*). The reaction mixture was incubated at 50 °C for 20 min. Reaction was stopped by adding 200 µL of trichloroacetic acid (10%, *w/v*). After centrifugation (8000× *g*, 10 min, 4 °C), 500 µL of the collected supernatant were added of 100 µL ferric chloride (0.1%, *w/v*) and 400 µL of demineralized water. After incubation (10 min, 25 °C), the absorbance of ferrous ion was measured at 700 nm. Ascorbic acid (1%, *w/v*) was used as a positive control [56].

The free radical scavenging activity against DPPH was measured according to the method described by Bersuder et al. [57]. In detail, 167 µL of sample (juice/methanolic extract of seeds/ethanolic extract of pudding) were mixed with 167 µL of DPPH (2.5 g/L) and 667 µL of methanol (80%, *v/v*). The reaction mixture was incubated in dark up to 120 min at 25 °C. The absorbance of the reaction mixture was measured at 517 nm at 0, 10, 20, 30, 60 and 120 min. Two positive controls were used: BHT (0.45 g/L in methanol 80%) and ascorbic acid (1%). A reaction mixture consisting just of 167 µL of DPPH and 833 µL of methanol (80%) was always inserted as the blank. The free radical scavenging activity was calculated as follows:

DPPH scavenging activity (%) = [(blank absorbance − sample absorbance)/blank absorbance] × 100 (1)

### 4.8. Sensory Analyses

Puddings, based on fermented zgougou juice (100 mL), flour (50 g) and sugar (25 g) were mixed while cooking (ca. 50 °C) for 20–30 min until getting a viscous and homogeneous pastry cream. Puddings were subjected to a panel test after 6 h from the end of cooking. The panel was composed of ten volunteers (4 male and 6 female with a mean age of 29-years-old, a range of 24–34 years old) from laboratory staff, which had been previously trained about the meaning of the sensory attributes and scores. Each pudding was identified by a code number and served, in a cup at 20 °C and under daylight illumination, in random order. Each panelist evaluated two spoons of pudding per thesis. The attributes were: appearance, color, odor, texture, sweet taste, sour taste, flavour, and overall acceptability. The score for each sensory attribute ranged from 1 (lowest) to 5 (highest) [58]. 

### 4.9. Statistical Analyses

One-way ANOVA was applied to the data from two biological replicates (analysed in triplicate for obtaining the results of culture-dependent microbiological analyses, VOC, antibacterial and antioxidant activities). Tukey’s procedure was used to carry out pair comparison of treatment means, using a *p* level of 0.05. In addition, the concentrations of VOC and the results about taxonomic structure of bacterial biome (assessed through culture-dependent and 16S rRNA high throughput sequencing analyses) were used for computing Spearman correlations, through Statistica version 12 for Windows.

## 5. Conclusion and Perspectives

The results of this study showed that the use, at laboratory scale, of autochthonous lactobacilli is a feasible biotechnological tool to outgrow undesired bacteria, thus improving the safety of juice from zgougou. The autochthonous lactobacilli widened the antibacterial spectrum of zgougou, and did not impair the intrinsic antioxidant activity of Aleppo pine’s seeds. Future studies should be undertaken to confirm the observed benefits at industrial scale.

## Figures and Tables

**Table 1:** pH values of zgougou inoculated with autochthonous lactic acid bacteria after 24 h of fermentation at 25 °C.

Microorganism	pH
*Lactobacillus paraplantarum* A1	4.65 ± 0.02^f^
*Lactobacillus plantarum* A2	4.65 ± 0.01^f^
*L. plantarum* A3	4.75 ± 0.03^e^
*Enterococcus faecalis* A4	4.84 ± 0.02^c^
*Ent. faecalis* A5	4.79 ± 0.02^de^
*Ent. faecalis* A6	4.89 ± 0.01^b^
*Ent. faecalis* A7	4.96 ± 0.01^a^
*Ent. faecalis* A8	4.83 ± 0.02^c^
*Ent. faecalis* A9	4.85 ± 0.03^c^
*Ent. faecalis* A10	4.76 ± 0.02^e^
*Ent. faecalis* A11	4.92 ± 0.03^ab^
*Ent. faecalis* A12	4.80 ± 0.01^d^
*Ent. faecalis* A13	4.85 ± 0.02^c^
*Ent. faecalis* A14	4.93 ± 0.02^a^

Data in the same column with different letters (^a–f^) are significantly different (*p* < 0.05).

**Table 2:** Alpha diversity indexes* of *Bacteria* (16S rRNA) found in the juice (J) and seeds (S) obtained after 24 h of zgougou fermentation (spontaneous, “LAB−”, or inoculated with autochthonous lactobacilli, “LAB+”).

Sample	Observed Species	Chao1	Shannon
J LAB-	17.0 ± 1.41^c^	17.3 ± 1.65^c^	1.71 ± 0.032^c^
S LAB-	21.5 ± 0.71^a^	21.5 ± 0.71^a^	2.06 ± 0.004^a^
J LAB+	3.0 ± 1.41^d^	3.0 ± 1.41^d^	0.44 ± 0.021^d^
S LAB+	20.0 ± 0.00^b^	20.0 ± 0.00^b^	1.91 ± 0.030^b^

* Values (mean of two replicates ± standard deviation) in the same column followed by different letters (a–d) are significantly different (*p* < 0.05).

**Table 3:** Relative abundance (%)* of bacterial OTUs found in the juice (J) and seeds (S) obtained after 24 h of zgougou fermentation (spontaneous, “LAB−”, or inoculated with autochthonous lactobacilli, “LAB+”).

Bacterial OTUs	J LAB−	S LAB−	J LAB+	S LAB+
*Bacillus cereus*	0.22 ± 0.02^b^	0.55 ± 0.34^b^	0^c^	0.99 ± 0.06^a^
*Enterococcus hirae*	0.87 ± 0.23^c^	1.60 ± 0.53^b^	0^c^	2.75 ± 0.48^a^
*Lactobacillus plantarum*	43.83 ± 1.39^b^	2.21 ± 0.95^d^	82.89 ± 1.15^a^	6.20 ± 0.19^c^
*Weissella confusa*	0^b^	0^b^	0^b^	9.34 ± 0.40^a^
*Lactococcus raffinolactis*	3.12 ± 0.34^b^	34.74 ± 0.82^a^	0^c^	0^c^
*Bacilli*	0.004 ± 0.00^b^	0.02 ± 0.00^a^	0^b^	0^b^
*Clostridiales*	0^a^	0.02 ± 0.00^a^	0^a^	0^a^
*Aeromonas sp*	1.43 ± 0.14^b^	2.75 ± 0.05^a^	0^d^	0.38 ± 0.10^c^
*Enterobacter sp*	28.43 ± 0.36^b^	37.68 ± 1.45^a^	0^d^	25.20 ± 0.47^c^
*Pantoea sp.*	0.01 ± 0.00^b^	0.08 ± 0.01^b^	0^b^	15.55 ± 1.09^a^
*Enterobacteriaceae*	0.71 ± 0.04^b^	1.28 ± 0.05^a^	0^c^	0.68 ± 0.03^b^
*Acinetobacter junii*	0.06 ± 0.02^b^	0.07 ± 0.00^b^	0^c^	0.14 ± 0.09^a^
*Acinetobacter radioresistens*	0^a^	0^a^	0^a^	0.01 ± 0.00^a^
*Acinetobacter sp*	1.92 ± 0.11^b^	5.31 ± 0.09^a^	0^d^	0.58 ± 0.18^c^
*Pseudomonas sp*	1.41 ± 0.03^a^	0.02 ± 0.01^c^	0^d^	0.64 ± 0.62^b^
*Gammaproteobacteria*	0^c^	0.06 ± 0.09^b^	0^c^	0.25 ± 0.01^a^
Other unclassified bacteria	17.93 ± 0.93^b^	13.54 ± 1.23^c^	17.1 ± 1.16^b^	37.22 ± 2.29^a^

* Values (mean of two replicates ± standard deviation) in the same row followed by different letters (a-d) are significantly different (*p* < 0.05).

**Table 4:** Concentration* (in mg/kg) of volatile organic compounds detected in the juice (J) and seeds (S) obtained after 24 h of zgougou fermentation (spontaneous, “LAB−”, or inoculated with autochthonous lactobacilli, “LAB+”).

Compounds	Odor	J LAB−	J LAB+	S LAB−	S LAB+
*Alcohols*					
Ethanol	Sweet, alcoholic, ripe apple	0.32 ± 0.142^a^	0.05 ± 0.001^b^	0.08 ± 0.021^A^	0.01 ± 0.003^B^
1-butanol 3-methyl	Fermented, malt, wine	0.03 ± 0.016^a^	0.01 ± 0.003^a^	Nd^A^	Nd^A^
3-butoxy-1-propanol	Nf	0.02 ± 0.009^a^	0.03 ± 0.004^a^	Nd^B^	0.10 ± 0.026^A^
1-hexanol	Green grass, flowery, woody, mild, sweet	0.04 ± 0.021^b^	0.09 ± 0.007^a^	0.14 ± 0.046^A^	0.19 ± 0.003^A^
3,5-octadien-2-ol	Bean-like	Nd^a^	Nd^a^	0.05 ± 0.016^A^	Nd^B^
1-nonanol	Ophrys	Nd^b^	0.02 ± 0.000^a^	Nd^B^	0.03 ± 0.008^A^
Benzyl alcohol	Boiled cherries, moss, roasted bread, rose	0.01 ± 0.007^a^	0.01 ± 0.002^a^	Nd^A^	Nd^A^
Phenylethyl alcohol	Rose-honey-like, wilted rose	0.02 ± 0.010^a^	0.01 ± 0.001^a^	0.04 ± 0.016^A^	0.03 ± 0.007^A^
*Aldehydes*					
Acetaldehyde	Fruity, floral, green apple, nut, penetrating	0.01 ± 0.005^a^	Nd^b^	0.14 ± 0.032^A^	0.01 ± 0.001^B^
Octane, 4-methyl-	Nf	0.01 ± 0.008^b^	0.02 ± 0.002^a^	Nd^B^	0.02 ± 0.003^A^
Butanal, 3-methyl-	Malty, roasty cucumber-like	Nd^a^	Nd^a^	0.02 ± 0.006^A^	Nd^B^
Pentanal	Nf	Nd^a^	Nd^a^	0.03 ± 0.011^A^	0.03 ± 0.014^A^
Hexanal	Green, grassy, tallow	Nd^a^	Nd^a^	0.26 ± 0.103^A^	0.46 ± 0.288^A^
Heptanal	Fatty, rancid, citrus, malty	Nd^a^	Nd^a^	0.02 ± 0.010^A^	0.03 ± 0.008^A^
Octanal	Aglaia, Cymbidium, Hydnora, Ophry	Nd^a^	Nd^a^	0.10 ± 0.052^A^	0.08 ± 0.037^A^
Nonanal	Fat, floral, green, lemon, paint	Nd^a^	Nd^a^	0.21 ± 0.096^A^	0.14 ± 0.059^A^
2-octenal	Fat, fish oil, green, nut, plastic	0.02 ± 0.010^a^	0.05 ± 0.001^a^	0.22 ± 0.080^A^	0.26 ± 0.067^A^
2-furaldehyde	Almond-like	0.03 ± 0.013^a^	0.04 ± 0.005^a^	0.22 ± 0.062^A^	0.29 ± 0.068^A^
Decanal	Stewed, burnt, green, waxy, floral, lemon	0.04 ± 0.017^a^	0.02 ± 0.002^a^	0.09 ± 0.032^A^	0.05 ± 0.013^A^
Benzaldehyde	Almond, caramel	0.02 ± 0.027^a^	0.03 ± 0.010^a^	0.10 ± 0.042^A^	0.10 ± 0.022^A^
2-nonenal	Fatty, tallowy, green	0.02 ± 0.009^a^	0.03 ± 0.004^a^	0.07 ± 0.028^A^	0.05 ± 0.012^A^
Decenal	tallow	Nd^a^	Nd^a^	0.07 ± 0.020^A^	0.07 ± 0.016^A^
2-undecenal	sweet	Nd^a^	Nd^a^	0.01 ± 0.005^A^	0.01 ± 0.002^A^
2,4-decadienal	seaweed	Nd^b^	0.04 ± 0.010^a^	0.09 ± 0.024^A^	0.11 ± 0.033^A^
Benzeneacetaldehyde	Berry, geranium, honey, nut, pungent	0.01 ± 0.007^a^	Nd^b^	Nd^B^	0.01 ± 0.003^A^
*Alkanes*					
Decane	Nf	0.02 ± 0.009^b^	0.06 ± 0.017^a^	0.04 ± 0.007^B^	0.07 ± 0.015^A^
Undecane	Nf	0.08 ± 0.029^a^	0.08 ± 0.006^a^	Nd^A^	Nd^A^
Nonane, 4,5-dimethyl-	Nf	0.03 ± 0.009^a^	0.03 ± 0.004^a^	Nd^A^	Nd^A^
Decane 2,4,6 dimethyl	Nf	0.19 ± 0.074^a^	0.24 ± 0.044^a^	0.15 ± 0.072^A^	0.15 ± 0.037^A^
Tricyclo[3.2.1.0(2,4)]octane,	Nf	0.03 ± 0.024^a^	0.04 ± 0.005^a^	Nd^A^	Nd^A^
Dodecane	Nf	0.01 ± 0.016^a^	0.01 ± 0.007^a^	Nd^A^	Nd^A^
Dodecane,4,6-dimethyl-	Nf	0.14 ± 0.062^a^	0.17 ± 0.021^a^	Nd^B^	0.03 ± 0.004^A^
Heptadecane, 2,6,10,15-tetramethyl-	Nf	0.01 ± 0.004^a^	0.01 ± 0.001^a^	Nd^A^	Nd^A^
Cyclopentane,1-ethenyl-3-ethyl-2-methyl	Nf	0.034 ± 0.013^b^	0.06 ± 0.007^a^	0.13 ± 0.039^A^	0.15 ± 0.038^A^
*Aromatic Compounds*					
Furan, 2-pentyl-	Butter, green bean, floral, fruity, mushroom, raw nuts	0.02 ± 0.007^a^	0.02 ± 0.003^a^	0.09 ± 0.034^A^	0.06 ± 0.016^A^
O-cymene	Citrus-like, solvent, gasoline	0.04 ± 0.018^a^	0.04 ± 0.005^a^	0.09 ± 0.016^A^	0.05 ± 0.024^A^
Estragole	Tarragon	Nd^a^	Nd^a^	0.03 ± 0.014^A^	0.01 ± 0.004^A^
*Carboxylic Acids*					
Acetic acid	Sour, nutty	Nd^b^	0.20 ± 0.023^a^	0.16 ± 0.030^B^	0.24 ± 0.055^A^
Butanoic acid	cheese, unpleasant	Nd^b^	0.01 ± 0.004^a^	Nd^A^	Nd^A^
Pentanoic acid	Sweet, rancid	Nd^a^	Nd^a^	0,03 ± 0.007^B^	0.05 ± 0.002^A^
Hexanoic acid (caproic acid)	Sweaty, cheesy, fatty, goat-like	0.03 ± 0.008^b^	0.19 ± 0.021^a^	0.63 ± 0.184^A^	0.81 ± 0.185^A^
Heptanoic acid	Cheese, fatty, sweaty	Nd^b^	0.02 ± 0.005^a^	0.07 ± 0.016^A^	0.08 ± 0.018^A^
Octanoic acid	Cheese, fatty, sweaty, soapy, chocolate-like	0.02 ± 0.00^b^	0.06 ± 0.015^a^	0.05 ± 0.056^A^	0.16 ± 0.040^A^
Nonanoic acid	Cheese, fatty, sweaty	0.05 ± 0.038 ^a^	0.09 ± 0.046^a^	0.16 ± 0.007^B^	0.26 ± 0.036^A^
*Esters*					
Ethyl acetate	Caramel, sweet, fruity, acid, buttery, pineapple	0.04 ± 0.018^a^	0.02 ± 0.002^b^	Nd^A^	Nd^A^
Hexanoic acid, ethyl ester	Apple peel-like, fruity	0.04 ± 0.000^a^	Nd^b^	Nd^A^	Nd^A^
Butanoic acid, pentyl ester	Nf	Nd^a^	Nd^a^	Nd^B^	0.02 ± 0.003^A^
Sulfurous acid, nonyl pentyl ester	Nf	Nd^a^	Nd^a^	0.07 ± 0.023^A^	Nd^B^
Octanoic acid, ethyl ester	Alcohol-like, Fruity, citrus-like	0.03 ± 0.010^a^	Nd^b^	Nd^A^	Nd^A^
Hexanoic acid, etenhyl ester	Nf	0.02 ± 0.010^a^	0.0 ± 0.003^a^	0.08 ± 0.025^A^	0.10 ± 0.019^A^
*Ketones*					
2-heptanone	Soapy, fruity, cinnamon	Nd^a^	Nd^a^	0.02 ± 0.004^B^	0.04 ± 0.004^A^
Acetoin	flowery, wet, butter, cream	0.15 ± 0.025^a^	0.11 ± 0.000^b^	0.89 ± 0.259^A^	0.07 ± 0.012^B^
2-octanone	Gasoline, mould, soap	Nd^b^	0.03 ± 0.009^a^	0.04 ± 0.008^A^	0.03 ± 0.005^A^
2-undecanone	fresh, green	Nd^a^	Nd^a^	Nd^B^	0.03 ± 0.007^A^
Acetophenone	Cheesy, sweet, almond, floral	0.02 ± 0.017	Nd^b^	Nd^A^	Nd^A^
*Others*					
1,3,5,7-cyclooctatetraene	Nf	0.01 ± 0.002^a^	Nd^a^	Nd^A^	Nd^A^
Indole	mothball, burnt	0.01 ± 0.018^a^	Nd^b^	Nd^A^	Nd^A^
*Phenols*					
Phenol, 2-methoxy-	Phenol,	0.43 ± 0.018^a^	0.02 ± 0.008^b^	0.54 ± 0.203^A^	0.06 ± 0.016^B^
Phenol	Phenol	0.05 ± 0.002^a^	0.01 ± 0.003^b^	0.08 ± 0.029^A^	Nd^B^
P-cresol	medicine, phenol, smoke	0.01 ± 0.000^a^	Nd^b^	Nd^A^	Nd^A^
Vanillin	Vanilla	Nd^a^	Nd^a^	Nd^B^	0.01 ± 0.004^A^
*Terpenes*					
α-pinene	Woody-spicy, oily, pine-like	3.45 ± 1.392^a^	3.21 ± 0.373^a^	2.63 ± 0.707^A^	2.56 ± 0.470^A^
β-pinene	resinous-piney, dry-terpenous	0.01 ± 0.008^a^	0.01 ± 0.005^a^	0.04 ± 0.023^A^	0.02 ± 0.005^A^
Camphene	Camphor	0.04 ± 0.017^a^	0.04 ± 0.003^a^	0.03 ± 0.009^A^	0.03 ± 0.004^A^
3-carene	lemon, resin	0.09 ± 0.036^a^	0.09 ± 0.007^a^	0.07 ± 0.017^A^	0.07 ± 0.013^A^
β-myrcene	weak citrus and lime-like	3.05 ± 1.216^a^	3.07 ± 0.497^a^	3.60 ± 0.898^A^	3.63 ± 0.730^A^
D-limonene	Citrus, Licorice, citrus, green, fruity	0.33 ± 0.145^a^	0.28 ± 0.027^a^	0.18 ± 0.061^A^	0.22 ± 0.040^A^
β-phellandrene	citrus-like, weak herbal-spicy	0.02 ± 0.035^a^	0.03 ± 0.006^a^	Nd^B^	0.06 ± 0.007^A^
4-methylisopropenyl-benzene	Nf	0.02 ± 0.010^a^	0.02 ± 0.003^a^	0.03 ± 0.016^A^	0.03 ± 0.006^A^
1,7,7-trimethylbicyclo [2.2.1]heptane-2,5-diol	Nf	Nd^b^	0.02 ± 0.001^a^	Nd^A^	Nd^A^
(+)-α-gurjunene	wood, balsamic	Nd^b^	0.01 ± 0.014^a^	0.05 ± 0.012^A^	Nd^B^
Bornyl acetate	Nf	Nd^b^	0.01 ± 0.001^a^	0.01 ± 0.004^B^	0.03 ± 0.008^A^
Caryophyllene	Dry, woody-spicy	0.28 ± 0.000^a^	0.05 ± 0.006^b^	0.30 ± 0.104^A^	0.07 ± 0.016^B^
Verbenol	Nf	0.02 ± 0.000^a^	0.01 ± 0.001^a^	0.03 ± 0.008^A^	0.03 ± 0.007^A^
Verbenone	Nf	0.05 ± 0.076^a^	0.04 ± 0.003^a^	0.12 ± 0.046^A^	0.06 ± 0.007^A^
Borneol	Pungent, mint	0.02 ± 0.018^a^	0.02 ± 0.000^a^	0.02 ± 0.007^A^	0.02 ± 0.013^A^
(-)-carvone	Nf	0.01 ± 0.008^a^	Nd^b^	0.03 ± 0.010^A^	0.01 ± 0.005^A^
α.-farnesene	wood, sweet	Nd^a^	Nd^a^	Nd^B^	0.05 ± 0.002^A^
Bornyl formate	Green	Nd^b^	0.01 ± 0.000^a^	Nd^B^	0.02 ± 0.013^A^

* Values (mean of six replicates ± standard deviation) in the same row with different superscript lowercase letters (a, b; J LAB and J LAB+) or uppercase letters (A, B; S LAB- and S LAB+) differ significantly (*p* < 0.05). Nd, not detected. Nf, not found in literature.

**Table 5:** Antibacterial activity^§^ of juice (J) and seeds (S) obtained after 24 h of fermentation (spontaneous, LAB−, or inoculated, LAB+, with autochthonous lactobacilli) of Aleppo pine’s seeds.

Target Bacterial Species	J LAB−	J LAB+	S LAB−	S LAB+
*Staphylococcus aureus*	−	+	−	+
*Staphylococcus epidermidis*	−	+	−	+
*Micrococcus luteus*	+	+	−	+
*Escherichia coli*	+	+	+	+
*Listeria monocytogenes*	−	++	+	+
*Enterococcus faecalis*	−	++	+	+
*Salmonella* Typhimurium	−	+	+	+
*Bacilus cereus*	+	+	−	+
*Vibrio parahaemolyticus*	−	++	+	+
*Aeromonas hydrophila*	−	+	−	−

^§^ −, no inhibition; +, 4 mm < diameter of inhibition halo <10 mm; ++, diameter of inhibition halo >10 mm.

## Supplementary Materials

Supplementary materials can be found at https://www.mdpi.com/2076-2607/8/1/29/s1. Figure S1. Relative abundance (%) of fungal OTUs classified at the highest possible taxonomic level found in the juice (J) and seeds (S) obtained after 24 h of zgougou fermentation (spontaneous, “LAB−”, or inoculated with autochthonous lactobacilli, “LAB+”). Table S1. Concentration (in mg/kg) of volatile organic compounds detected in the juice (J) and seeds (S) before zgougou fermentation. Table S2. Correlations between main bacterial OTUs and VOCs detected in juice and seeds obtained after 24 h of zgougou fermentation.
