# Supplementary material for: Use of Autochthonous Lactobacilli to Increase the Safety of Zgougou
Source: Microorganisms. 2019 Dec 22;8(1):29. doi: 10.3390/microorganisms8010029 (PMC7023124; doi:10.3390/microorganisms8010029)
Supplement: Supplementary file 1 [file microorganisms-08-00029-s001.pdf]

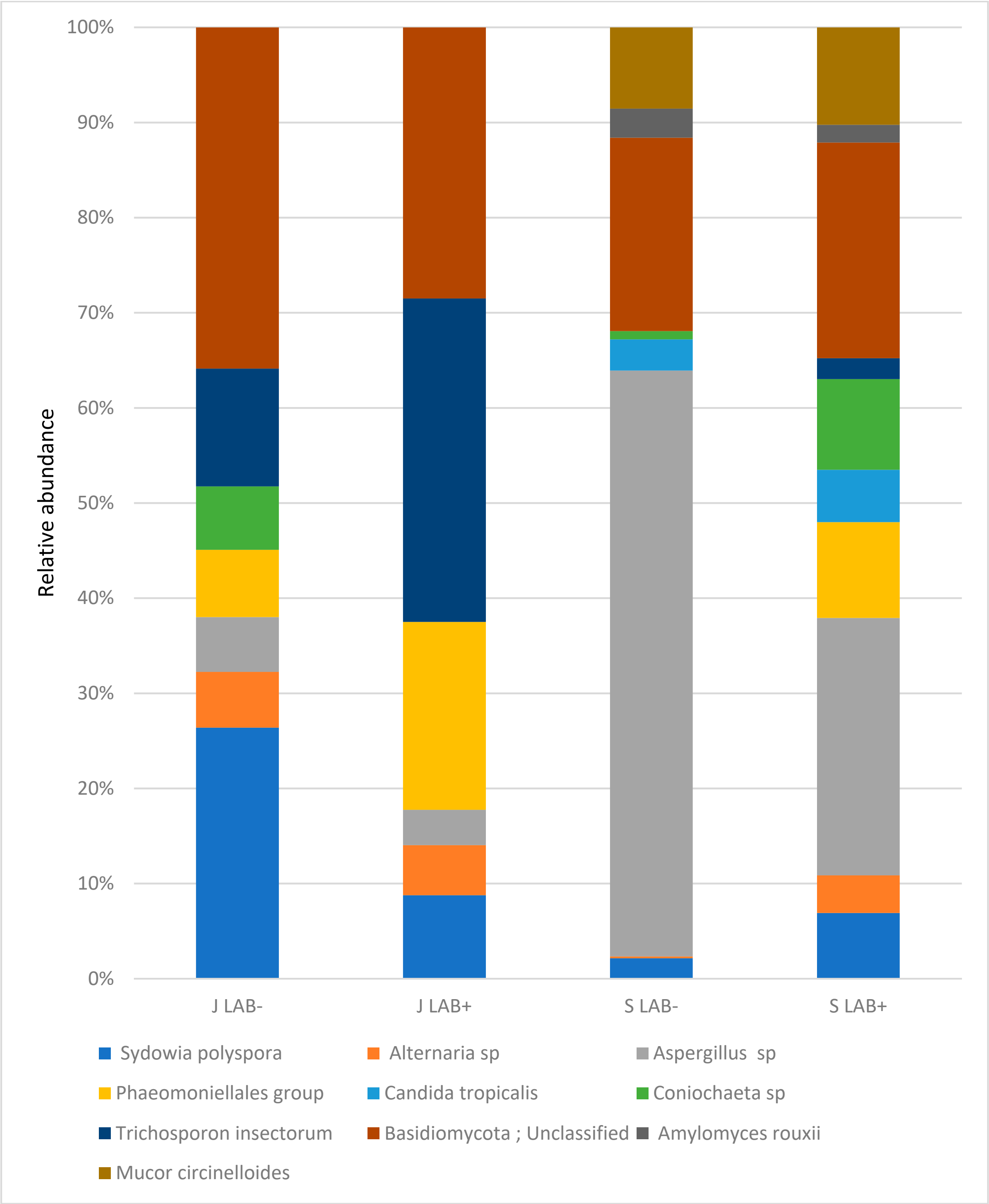

Figure S1. Relative abundance (%) of fungal OTUs classified at the highest possible taxonomic level found in the juice (J) and seeds (S) obtained after 24 h of zgougou fermentation (spontaneous, "LAB-", or inoculated with autochthonous lactobacilli, "LAB+").

Table S1. Concentration (in mg/kg) of volatile organic compounds detected in the juice (J) and seeds (S) before zgougou fermentation.

Compounds	Odor	J	S
Alcohols			
1-hexanol	Green grass, flowery, woody, mild, sweet	0.01	Nd
3,5-octadien-2-ol	Bean-like	0.02	0.16
Benzyl alcohol	Boiled cherries, moss, roasted bread, rose	0.01	Nd
Phenylethyl alcohol	Rose-honey-like, wilted rose	0.01	0.01
Percentage (%)		0.61	0.95
Aldehydes			
Acetaldehyde	Fruity	0.01	0.03
Octane, 4-methyl-	Nf	0.02	0.04
Butanal, 2-methyl-	Almond, malty	Nd	0.01
Butanal, 3-methyl-	Malty, roasty cucumber-like	Nd	0.06
Pentanal	Nf	Nd	0,14
Hexanal	Green, grassy, tallow	0.13	1.47
Heptanal	Fatty, rancid, citrus, malty	Nd	0.11
Octanal	Aglaia, Cymbidium, Hydnora, Ophry	Nd	0.32
Nonanal	Fat, floral, green, lemon, paint	Nd	0.42
2-octenal	Fat, fish oil, green, nut, plastic	0.05	0.59
2-furaldehyde	Almond-like	0.05	0.32
Decanal	Stewed, burnt, green, waxy, floral, lemon	0.04	0.14
Benzaldehyde	Almond, caramel	0.03	0.15
2-nonenal	Fatty, tallowy, green	0.02	0.09

Benzeneacetaldehyde	Berry, geranium, honey, nut, pungent	Nd	0.14
Percentage (%)		3.56	21.79
Alkanes			
Hexane, 2,3,4-trimethyl-	Nf	0,05	0,09
Tridecane	Nf	0.05	0.10
Decane	Nf	0.09	0.20
Nonane, 4,5-dimethyl-	Nf	0.08	0.17
Decane 4-methyl	Nf	0.02	0.06
Decane 2,4,6 dimethyl	Nf	0.42	0.87
Dodecane	Nf	0.13	0.42
Dodecane, 4,6-dimethyl-	Nf	0.19	0.36
Heptadecane, 2,6,10,15-tetramethyl-	Nf	0.06	0.13
Tetradecane	Nf	0.04	0.14
Cyclopentane,1-ethenyl-3-ethyl-2-methyl	Nf	0.02	0.11
Percentage (%)		11.61	14.47
Aromatic Compounds			
Furan, 2-pentyl-	Butter, green bean, floral, fruity, mushroom, raw nuts	0.03	0.13
O-cymene	Citrus-like, solvent, gasoline	0.05	0.07
Percentage (%)		0.73	1.10
Carboxylic acids			
Hexanoic acid (caproic acid)	Sweaty, cheesy, fatty, goat-like	Nd	0.26
Heptanoic acid	Cheese, fatty, sweaty	Nd	0.03
Octanoic acid	Cheese, fatty, sweaty, soapy	Nd	0.09
Nonanoic acid	Cheese, fatty, sweaty	Nd	0.18
Percentage (%)		0	3.03
Esters			

Octanoic acid, ethyl ester	Alcohol-like, Fruity, citrus-like	Nd	0.01
Hexanoic acid, etenhyl ester	Nf	Nd	0.16
Percentage (%)		0	0.92
Ketones			
2-heptanone	Soapy, fruity, cinnamon	Nd	0.06
Acetophenone	Cheesy, sweet, almond, floral	Nd	0.10
Percentage (%)		0	0.86
Others			
Cyclooctene, 3-butoxy	Nf	0.04	0.18
Percentage (%)		0.38	0.99
Phenols			
Phenol, 2-methoxy-	Phenol	0.01	0.04
Phenol	Phenol	Nd	0.05
Percentage (%)		0.09	0.52
Terpenes			
α-pinene	Woody-spicy, oily, pine-like	3.69	4.55
Camphene	camphor	0.06	0.08
3-carene	lemon, resin	0.09	0.10
.β.-myrcene	weak citrus and lime-like	3.63	4.19
D-limonene	Citrus, Licorice, citrus, green, fruity	0.42	0.46
β-phellandrene	citrus-like, weak herbal-spicy	0.06	0.02
4-Methylisopropenylbenzene	Nf	0.13	0.33
Bornyl acetate	Nf	0.01	0.02
Caryophyllene	Dry, woody-spicy	0.04	0.12
Linalyl acetate	sweet, fruit	Nd	0.03
Verbenol	Nf	0.02	0.05
Verbenone	Nf	0.04	0.15

Borneol	Pungent, mint	0.01	0.06
(-)-carvone	Nf	Nd	0.02
Percentage (%)		83.03	55.38

Nd, not detected. Nf, not found in literature.

Table S2. Correlations between main bacterial OTUs and VOCs detected in juice and seeds obtained after 24 h of zgougou fermentation.

	B. cereus	*E. hirae*	*L. plantarum*	*W. confusa*	*Lc. raffinolactis*	*Bacilli*	*Aeromonas* sp.	*Enterobacter* sp.	*Pantoea* sp.	*Enterobacteriaceae*	*A. junii*	*Acinetobacter* sp.	*Pseudomonas* sp.	*Gamma-proteobacteria*
Ethanol	-0.26	-0.18	0.20	-0.17	-0.42	-0.31	-0.01	0.15	-0.18	-0.05	0.00	-0.18	0.95*	-0.31
3-butoxy-1-propanol	0.71*	0.70*	-0.31	0.96*	-0.59	-0.62	-0.55	-0.01	0.96*	-0.14	0.77*	-0.56	0.33	0.87*
1-hexanol	0.88*	0.82*	-0.68	0.80*	0.18	0.09	-0.05	0.24	0.80*	0.30	0.72*	0.06	-0.41	0.90*
3,5-octadien-2-ol	0.17	0.17	-0.56	-0.33	1.00*	0.98*	0.87*	0.61	-0.33	0.78*	0.03	0.94*	-0.50	-0.10
1-nonanol	0.48	0.42	0.00	0.83*	-0.60	-0.68	-0.78*	-0.45	0.83*	-0.48	0.41	-0.72*	-0.18	0.75*
Acetaldehyde	0.22	0.23	-0.62	-0.30	1.00*	0.99*	0.91*	0.69	-0.30	0.84*	0.10	0.96*	-0.42	-0.07
Octane, 4-methyl-	-0.11	-0.18	0.58	0.39	-0.79*	-0.85*	-0.97*	-0.88*	0.39	-0.89*	-0.16	-0.92*	-0.21	0.23
Butanal,3-methyl-	0.17	0.17	-0.56	-0.33	1.00*	0.98*	0.87*	0.61	-0.33	0.78*	0.03	0.94*	-0.50	-0.10
2-octenal	0.92*	0.89*	-0.86*	0.69	0.41	0.35	0.25	0.51	0.69	0.57	0.78*	0.34	-0.33	0.84*
2,4-decadienal	0.81*	0.75*	-0.69	0.65	0.36	0.27	0.10	0.28	0.65	0.38	0.62	0.22	-0.55	0.79*
Benzeneacetaldehyde	0.51	0.56	-0.31	0.65	-0.54	-0.50	-0.25	0.28	0.65	0.06	0.71*	-0.36	0.83*	0.54
Decane	0.45	0.37	-0.03	0.73*	-0.37	-0.47	-0.66	-0.46	0.72*	-0.41	0.30	-0.55	-0.48	0.69
Dodecane,4,6-dimethyl-	-0.84*	-0.83*	0.95*	-0.45	-0.66	-0.62	-0.57	-0.75*	-0.45	-0.82*	-0.73*	-0.63	0.26	-0.65
Cyclopentane,1-ethenyl-3-ethyl-2-methyl	0.87*	0.83*	-0.76*	0.70	0.35	0.27	0.13	0.37	0.70*	0.45	0.71*	0.24	-0.44	0.84*
Acetic acid	0.41	0.32	-0.02	0.65	-0.28	-0.38	-0.61	-0.46	0.65	-0.39	0.23	-0.48	-0.59	0.64
Pentanoic acid	0.98*	0.95*	-0.82*	0.84*	0.19	0.13	0.08	0.45	0.84*	0.46	0.89*	0.14	-0.16	0.95*
Hexanoic acid	0.92*	0.88*	-0.78*	0.76*	0.28	0.21	0.10	0.39	0.76*	0.44	0.78*	0.19	-0.35	0.89*
Heptanoic acid	0.86*	0.81*	-0.76*	0.68	0.37	0.29	0.15	0.37	0.68	0.45	0.69	0.26	-0.47	0.82*

Octanoic acid	0.82*	0.77*	-0.45	0.95*	-0.25	-0.33	-0.42	-0.01	0.95*	-0.03	0.73*	-0.34	-0.18	0.96*
Nonanoic acid	0.92*	0.88*	-0.70*	0.87*	0.09	0.01	-0.09	0.27	0.87*	0.30	0.81*	0.00	-0.27	0.95*
Ethyl acetate	-0.72*	-0.66	0.65	-0.53	-0.46	-0.37	-0.17	-0.27	-0.53	-0.40	-0.51	-0.30	0.65	-0.69
Hexanoic acid, ethyl ester	-0.34	-0.25	0.18	-0.33	-0.25	-0.14	0.16	0.23	-0.34	0.05	-0.09	-0.01	0.89*	-0.44
Butanoic acid, pentyl ester	0.85*	0.83*	-0.49	1.00*	-0.37	-0.42	-0.41	0.10	1.00	0.02	0.84	-0.38	0.12	0.97*
Sulfurous acid, nonyl pentyl ester	0.17	0.17	-0.56	-0.33	1.00*	0.98*	0.87*	0.61	-0.33	0.78*	0.03	0.94*	-0.50	-0.10
Octanoic acid, ethyl ester	-0.34	-0.25	0.18	-0.33	-0.25	-0.14	0.16	0.23	-0.34	0.05	-0.09	-0.01	0.89*	-0.44
2-heptanone	0.97*	0.94*	-0.85*	0.80*	0.26	0.20	0.14	0.49	0.80*	0.52	0.87*	0.21	-0.20	0.92*
Acetoin	0.11	0.11	-0.52	-0.40	1.00*	0.99*	0.90*	0.62	-0.40	0.78*	-0.02	0.96*	-0.46	-0.17
2-octanone	0.34	0.26	-0.31	0.18	0.51	0.41	0.13	-0.02	0.18	0.18	0.08	0.29	-0.92*	0.34
Acetophenone	-0.34	-0.25	0.18	-0.33	-0.25	-0.14	0.16	0.23	-0.34	0.05	-0.09	-0.01	0.89*	-0.44
Indole	-0.34	-0.25	0.18	-0.33	-0.25	-0.14	0.16	0.23	-0.34	0.05	-0.09	-0.01	0.89*	-0.44
Phenol,2-methoxy-	-0.04	0.03	-0.45	-0.52	0.76*	0.83*	0.96*	0.80*	-0.52	0.82*	0.02	0.90*	0.22	-0.37
Phenol	-0.18	-0.13	-0.33	-0.66	0.83*	0.89*	0.95*	0.67	-0.66	0.74*	-0.17	0.92*	0.02	-0.50
P-cresol	-0.34	-0.25	0.18	-0.33	-0.25	-0.14	0.16	0.23	-0.34	0.05	-0.09	-0.01	0.89*	-0.44
Vanillin	0.85*	0.83*	-0.49	1.00*	-0.37	-0.42	-0.41	0.10	1.00*	0.02	0.84*	-0.38	0.12	0.97*
β-phellandrene	0.45	0.43	0.02	0.85*	-0.81*	-0.84*	-0.80*	-0.34	0.84*	-0.47	0.50	-0.81*	0.29	0.70*
1,7,7-trimethylbicyclo[2.2.1]heptane-2,5-diol	-0.68	-0.75*	0.87*	-0.33	-0.37	-0.42	-0.62	-0.94*	-0.34	-0.85*	-0.78*	-0.55	-0.52	-0.44
(+)-α-gurjunene	-0.03	-0.05	-0.32	-0.46	0.94*	0.91*	0.73*	0.36	-0.45	0.56	-0.21	0.83*	-0.69	-0.24
Bornyl acetate	0.83*	0.78*	-0.51	0.90*	-0.10	-0.18	-0.31	0.04	0.90*	0.07	0.71*	-0.21	-0.32	0.94*

Caryophyllene	-0.06	0.02	-0.42	-0.52	0.70*	0.77*	0.93*	0.79*	-0.52	0.79*	0.02	0.85*	0.30	-0.39
(-)-carvone	0.51	0.54	-0.86*	-0.01	0.89*	0.90*	0.90*	0.90*	-0.01	0.98*	0.45	0.93*	-0.13	0.21
α.-farnesene	0.85*	0.83*	-0.49	1.00*	-0.37	-0.42	-0.41	0.10	1.00*	0.02	0.84*	-0.38	0.12	0.97*
Bornyl formate	0.28	0.21	0.20	0.69	-0.64	-0.72*	-0.86*	-0.63	0.69	-0.64	0.20	-0.78*	-0.28	0.58

*Significant correlations found at a false discovery rate (FDR) of < 0.05. *B.*, *Bacillus*; *E.*, *Enterococcus*; *L.*, *Lactobacillus*; *W.*, *Weissella*; *Lc.*, *Leuconostoc*; *A.*, *Aeromonas*
